# Harnessing Information Thermodynamics: Conversion of DNA Information into Mechanical Work in RNA Transcription and Nanopore Sequencing

**DOI:** 10.3390/e26040324

**Published:** 2024-04-11

**Authors:** Tatsuaki Tsuruyama

**Affiliations:** 1 Department of Discovery Medicine, Graduate School of Medicine, Kyoto University, Kyoto 606-8501, Japan; tsuruyam@ddm.med.kyoto-u.ac.jp; Tel.: +81-75-366-7417; 2 Department of Physics, Graduate School of Science, Tohoku University, Sendai 980-8578, Japan; 3 Department of Clinical Laboratory, Graduate School of Health Sciences, Kyoto Tachibana University, Kyoto 607-8175, Japan; 4 Kitano Medical Institute, Kitano Hospita, Osaka 530-8480, Japan

**Keywords:** mutual information, transcription, RNA polymerase, free energy change, fluctuation theorem, nanopore sequencing

## Abstract

Recent advancements in information thermodynamics have revealed that information can be directly converted into mechanical work. Specifically, RNA transcription and nanopore sequencing serve as prime examples of this conversion, by reading information from a DNA template. This paper introduces an information thermodynamic model in which these molecular motors can move along the DNA template by converting the information read from the template DNA into their own motion. This process is a stochastic one, characterized by significant fluctuations in forward movement and is described by the Fokker–Planck equation, based on drift velocity and diffusion coefficients. In the current study, it is hypothesized that by utilizing the sequence information of the template DNA as mutual information, the fluctuations can be reduced, thereby biasing the forward movement on DNA and, consequently, reducing reading errors. Further research into the conversion of biological information by molecular motors could unveil new applications, insights, and important findings regarding the characteristics of information processing in biology.

## 1. Introduction

The integration of nonequilibrium thermodynamics into biophysics has markedly progressed, covering areas such as membrane transport [1,2,3], signal transduction [4,5], and the study of molecular motors [6,7,8,9,10]. This field has seen significant theoretical advancements, notably the discovery of the fluctuation theorem [11,12,13,14], which have enabled mechanical measurements to determine the forces propelling molecular motors [6] and to estimate torque in singular instances, including experiments on F1-adenosine triphosphatase [7,8,9]. RNA polymerase (RNAP), a molecular motor of considerable interest, translocates along DNA to synthesize RNA precursors, driven predominantly by the free energy change from the hydrolysis of ribonucleotide triphosphates (rNTP) [15,16]. Notably, reports on the free energy changes associated with RNAP translocation have varied widely, ranging from −1.3 *k_B_T* to 3.4 *k_B_T* [17,18,19], a variance that has, yet, to be adequately explained [17,18,19,20], thus highlighting a knowledge gap in our understanding of RNAP mechanics.

Previously, we reported on an information thermodynamics model related to the movement of RNAP [21]. In that report, we detailed the kinetics of RNAP and described a mechanism through which mutual information, derived from the sequence information of the template DNA, is converted into work for RNAP’s own movement. This framework within information thermodynamics explains that the extent to which fluctuating stochastic processes and contributions of mutual information are incorporated in the free energy change results in the variations in the value.

This article introduces an advanced model for the transcription process by RNA polymerase (RNAP), based on the foundation of experimental studies [22]. The model employs the Fokker–Planck equation, incorporating drift and diffusion coefficients characteristic of molecular motors, to elucidate the stochastic behavior of RNAP movement. Furthermore, this framework is extended to explore the thermodynamic properties of molecular motors in nanopore sequencing. Nanopore sequencing represents an innovative technology for deciphering long sequences of DNA, by passing individual DNA molecules through a nanopore embedded in a synthetic membrane. As each deoxyribonucleotide triphosphate (dNTP = dATP, dTTP, dCTP, dGTP) traverses the nanopore, it induces distinctive perturbations in the ionic current, enabling the identification of the specific dNTP type. This technique offers a means to directly sequence DNA and RNA molecules without the need for amplification via polymerase chain reaction (PCR) or complex chemical reactions. Fundamentally, nanopore sequencing is based on the principle of detecting changes in electrical conductivity caused by nucleic acid molecules passing through nanoscale pores embedded in an electrically insulating membrane. In nanopore sequencing machinery, a type of molecular motor, known as helicase, plays a role in unwinding double-stranded DNA and supplying it in a single-stranded form to the nanopore. In this process, the helicase moves along the DNA strand. The molecular motor bears a resemblance to RNAP moving along template DNA. Therefore, we propose a unified theoretical model to understand these mechanisms cohesively [23,24].

**Figure 1:**
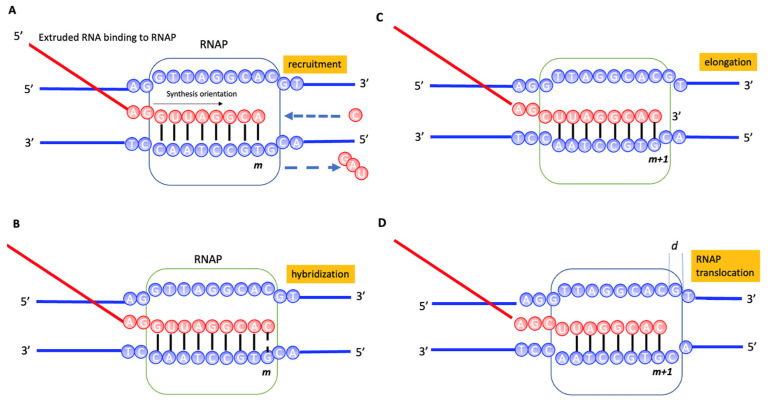
RNAP translocation model. The figure represents a transcription apparatus, where the thin rectangle symbolizes RNA polymerase (RNAP). The thick red line represents the RNA precursor, composed of ribonucleotides rNTP (rGTP, rCTP, rATP, rUTP) denoted by the letters G, C, A, U. The thick blue line signifies the template DNA, composed of deoxyribonucleotides dNTP (dGTP, dCTP, dATP, dTTP) represented by the letters G, C, A, T. Detailed naming for rNTP and dNTP is shown in the Abbreviation section. *m* and *m* + 1 denote the position of dNTPs. (**A**) Ribonucleotide recruitment: RNA polymerase (RNAP) accurately reads dGTP on the template DNA. It then selectively incorporates rCTP, while excluding non-complementary rNTPs. (**B**) Ribonucleotide hybridization: the incorporated rCTP forms hydrogen bonds with dGTP at the RNA reading edge. (**C**) Elongation: at this stage, the incorporated rCTP is added to the growing RNA precursor. (**D**) Forward bias in RNAP motion: the incorporation of rCTP prevents any arbitrary backward movement of RNAP, inducing a forward bias in its motion. Thus, the nucleotide type, in this case, dGTP, is represented as mutual information, converting into the translocation work of RNAP.

The commonality between nanopore sequencing and transcription processes involving molecular motors lies in the fact that during each step of movement between two dNTPs, the molecular motor is repeatedly reset to its pre-movement state (refer to Figure 1). This phenomenon originates from the repetitive structure of DNA, which is a double helix. Furthermore, although to varying degrees, both RNA polymerase (RNAP) and nanopore motor proteins exhibit fluctuations in their direction of movement, ultimately demonstrating a stochastic bias towards forward motion. To account for this bias, we previously reported that RNAP can function as a Maxwell’s demon, capable of converting information encoded in the DNA sequence into directed motion [23,24]. In this paper, we explore the possibility of extending this concept to the relative movement of motor molecules along template DNA in nanopore sequencing.

## 2. Methods

### 2.1. Transcription System Model

**Figure 2:**
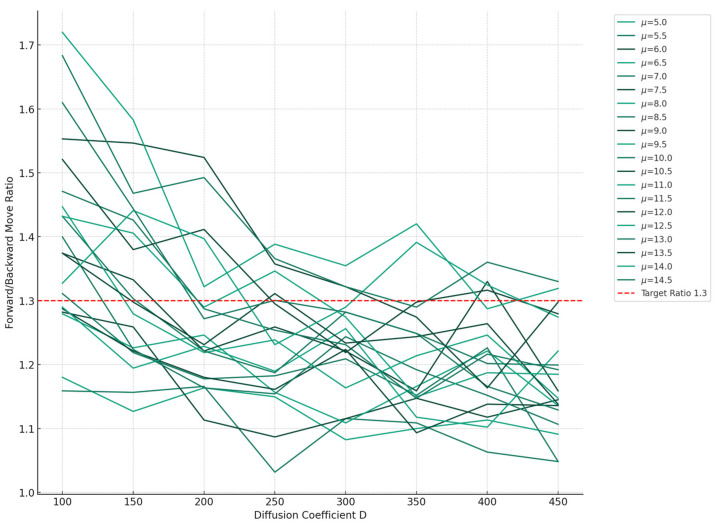
Plot of the ratio of forward to backward movement of RNAP against the diffusion coefficient. The drift velocity was varied in increments of 0.5, ranging from 5.0 to 14.5. The red dashed line represents the ratio of forward-to-backward movement = 1.3, based on experimental data. The closest approximation to a movement ratio of 1.3 was observed at a drift velocity (*μ*) of 13.0 and a diffusion coefficient (*D*) of 300, where the movement ratio was approximately 1.30.

**Figure 3:**
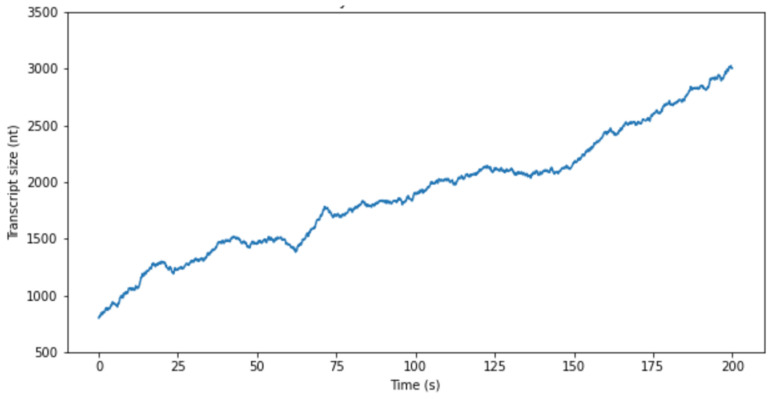
A plot of RNAP movement on the template T7 promoter of the λ phage. The model was predicated on a stochastic random walk paradigm. The abscissa delineates the duration of movement, whereas the ordinate reflects the quantum of base pairs traversed along the template DNA. Computations for the plot were underpinned by previously documented experimental outcomes, wherein RNA Polymerase (RNAP) was observed to traverse approximately 3 kB of DNA within a span of 200 s [18]. Within the framework of this simulation, the ratio of *μ* to *D* was established at 13/300.

**Figure 4:**
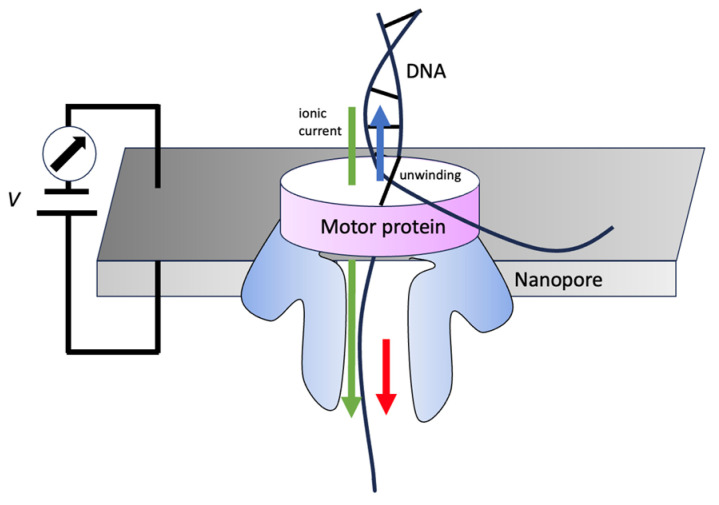
The motor protein helicase in nanopore sequencing. The upward blue arrow indicates the relative movement of the motor protein along the DNA, while the downward red arrow represents the actual movement of the DNA through the nanopore. A downward green arrow represents ionic current.

The molecular mechanism of RNAP involves intricate processes where RNAP reads the DNA template and synthesizes RNA strands, commencing with the precise recognition of promoter sequences to initiate transcription. This is followed by the elongation phase, where RNAP exhibits remarkable processivity, adding ribonucleotides in a template-directed manner, while ensuring transcriptional fidelity through nucleotide selection and proofreading. The activity is finely modulated by various transcription factors and co-factors that influence initiation, elongation, and termination phases. Structural dynamics play a crucial role, with conformational changes within RNAP being pivotal throughout the transcription cycle [17,19,25,26,27,28]. The transcription proceeds in four steps, as follows: (1) recruitment of dNTP to be transcribed (Figure 1A), (2) hybridization of the recruited rNTP with the template DNA (Figure 1B), (3) RNA precursor elongation by adding the rNTP to the RNA precursor bound to the RNAP (Figure 1C), and (4) the entire RNAP translocation (Figure 1D).

Thus, the nucleotide type, in this case, dGTP, is represented as mutual information, converting into the translocation work of RNAP.

When analyzing the movement of RNAP with short template DNA, the master equation, which represents the time evolution of the probability of each state, proves to be effective. If the probability of RNAP moving from position *x* to position *x* + 1 in the next time step is *P_f_*, and the probability of moving from position *x* to position *x* − 1 is *P_b_*, the master equation can be described as follows. The temporal evolution of the probability *P*(*x*,*t*) that RNAP is located at position *x* and time *t* is described by the following expression:

(1) $$\frac{\partial P\left(x,t\right)}{\partial t}=P_{f}P\left(x-1,t\right)+P_{b}P(x+1,t)-(P+P_{b})P(x,t)$$

Here, the first term on the right side represents an increase in probability due to moving from *x* − 1 to *x*, the second term represents an increase from *x* + 1 to *x*, and the third term represents a decrease from *x* to other positions. *P_f_* (*x*,*t*) and *P_b_*(*x*,*t*) represent the probability distribution functions for RNA polymerase moving forward and backward, respectively, at any time *t* and position *x*. Meanwhile, in the continuous limit for long template DNA, the probability *P*(*x*,*t*) that RNAP is located at position *x* is as follows:

(2) $$\frac{\partial P\left(x,t\right)}{\partial t}=D\frac{\partial^{2}P\left(x,t\right)}{\partial x^{2}}-\mu \frac{\partial P\left(x,t\right)}{\partial x}$$

where *D* is the diffusion coefficient and *μ* is the drift velocity. If the drift velocity and the diffusion coefficient are constant, the probability density function follows a normal distribution. This process is similar to Brownian motion and if the initial position is known as *x* = 0, the distribution of RNAP moving forward at any time *t* and position *x* is:

(3) $$P_{f}\left(x,t\right)\propto exp\left(-\frac{{\left(x-\mu t\right)}^{2}}{4Dt}\right)$$

The coordinate of the leading edge reached by RNAP is denoted by *L* = *Nd* and the probability density function for the rare event of reverse motion is given as follows:

(4) $$P_{b}\left(x,t\right)\propto exp\left(-\frac{{\left(x-L-\mu t\right)}^{2}}{4Dt}\right)$$

In a report measuring the fluctuational movement of RNAP on the template DNA, the ratio of forward to backward fluctuations was found to be approximately 1.3 [18]. In Equations (3) and (4), which models movements akin to Brownian motion, we conducted simulations by varying the drift velocity, *μ*, and the diffusion coefficient, *D*, to achieve a forward-to-backward fluctuation ratio of 1.3, as shown in Figure 2.

By comparing plots from an experimental study [18] (see Figure 2) and simulation (Figure 3), it was found that in Equation (3), *μ/D* is approximately 1/30, with its logarithmic value being 0.03.

The low ratio indicates that the thermodynamic force promoting motion is small, suggesting that random fluctuations dominate the movement. Thus, the thermodynamic driving force is not optimized for precise transcription. Consequently, considering the contribution of drift from mutual information derived from the nucleotide sequence of the template DNA implies that the information from the sequence acts as a “guide” for the movement direction of RNAP, reducing fluctuations in movement and directing RNAP to proceed forward during the reading of the DNA template, thereby instructing more accurate transcription by RNAP. As a result, the fidelity of transcription is enhanced.

To calculate the forces involved in motion, we use the probability density functions based on Equations (3) and (4), define the transition probabilities as *R_f_* and *R_b_* according to the fluctuation theorem, assume that the basic interval *d* is approximately 0.39 nm, the transcription position at *m*th bases with *x* = *md* (1 ≤ *m* ≤ *N*), and, further, taking into account experimental data that the ratio of forward to backward motions is about 1.3, log *R_f_/R_b_*~0.8, and the entropy change involved in motion can be calculated as follows, by taking the logarithm of the ratio of probability density functions according to the fluctuation theorem:

(5) $$∆S\left(x,t\right)=\log\frac{R_{f}{(x,t)P}_{f}(x,t)}{R_{b}{(x,t)P}_{b}(x,t)}$$

Substituting Equations (3) and (4) into Equation (5) and taking the limit as *t* approaches infinity, we have:

(6) $$∆S(x,t)=k_{B}\left(\frac{\mu L}{2D}+\log\frac{R_{f}(x,t)}{R_{b}(x,t)}\right)$$

And we introduced the entropy change per unit length of the template DNA:

(7) $$∆s(x,t)=k_{B}\left(\frac{\mu }{2D}+L^{-1}\log\frac{R_{f}(x,t)}{R_{b}(x,t)}\right)$$

In the steady state, the first term on the right-hand side is determined to be constant, whereas the second term accounts for the effects of fluctuations. The second term on the right-hand side corresponds to the heat absorbed by the heat bath in contact with the system when the RNAP moves from *m* to *m* + 1, provided that the system is in or not far from equilibrium, as described by the local detailed balance formula. In this model, the movement along the template DNA is divided by the distance *d* between dNTPs, assuming that RNAP reads *m* nucleotides, reaching *x* = *md*. We can define the free energy change required for the transition from per *L*:

(8) $$∆g_{m}=-k_{B}T∆s(m)$$

In the above, $∆s\left(m\right)$ = $∆s\left(x,t\right)$. Taking the average of the left hand, the equation can be expressed as:

(9) $$∆G_{m}=\langle ∆g_{m}\rangle =-k_{B}T\langle \frac{\mu }{2D}+L^{-1}\log\frac{R_{f}(m)}{R_{b}(m)}\rangle$$

In the above, $R_{f}\left(x,t\right)$ = $R_{f}\left(m\right)$ and $R_{b}\left(x,t\right)$ = $R_{b}\left(m\right)$. From Equation (9),

(10) $$\langle \Delta \Delta G_{m}\rangle =-k_{B}T\left(\langle \frac{\mu }{2D}+L^{-1}\log\frac{R_{f}(m)}{R_{b}(m)}\rangle -\langle \frac{\mu }{2D}+L^{-1}\log\frac{R_{f}(m+1)}{R_{b}(m+1)}\rangle \right)\;\leq -k_{B}T\langle L^{-1}\log\frac{R_{f}\left(m\right)R_{b}\left(m+1\right)}{{R_{f}\left(m+1\right)R}_{b}\left(m\right)}\rangle \;<0$$

In the equation above, we assume that the movement of RNAP does not progress as *m* increases, and we used equations *R_f_*(*m*) > *R_f_* (*m* +1) and *R_b_* (*m* + 1) > *R_b_* (*m*). This Equation (10), under the assumption that the transition probability *R*(*m*) is independent of *m*, can explain previous reports suggesting that 〈ΔΔ*G*〉 approaches a value close to zero. However, it cannot account for reports that indicate a positive value for 〈ΔΔ*G*〉 [17,18,19].

### 2.2. Information Thermodynamics of Gaining Mutual Information

In this study, we propose a model wherein RNAP utilizes the base sequence information from the template DNA as mutual information for its translocation. The mutual information arises as RNAP, positioned between (*m* − 1)*d* and *md*, acquires information about whether the incoming rNTPs can hybridize with dNTPs at position *m*, also denoting the degree of polymerization of dNTPs at the *N* position. If an rNTP is capable of hybridizing, it is incorporated into the transcription complex; otherwise, it is not incorporated (refer to Figure 1A). For instance, in the transcription initiation from the extensively studied T7 promoter DNA sequence, the probability of the appearance of dGTP is estimated to be 0.22 and the probability that the corresponding rCTP is incorporated is also estimated to be 0.22 (refer to Table 1). The step where RNAP identifies the type of rNTP is akin to a selection measurement where one out of the four types of rNTPs is chosen, reminiscent of Maxwell’s demon performing a binary measurement of particle velocities exceeding a specific threshold [25]. Here, *P_m_* denotes the probability of the rNTP to be selected, when RNAP is at position *m* = dATP, dUTP, dCTP, or dGTP. In this case, the mutual information is given by:

(11) $$I_{m}={-k}_{B}\Sigma_{m}P_{m}\log P_{m}$$

(12) $$i_{m}={-k}_{B}logP_{m}$$

In the information thermodynamic framework, the RNAP translocation step is considered as the feedback step from the dNTP in the template DNA (Figure 1D). The mutual information feedback is defined as follows:

(13) $$I_{m}={-k}_{B}\Sigma_{m}{P\prime }_{m}\log{P\prime }_{m}$$

(14) $$i_{m}={-k}_{B}log{P\prime }_{m}$$

Here, *P*′*_m_* denotes the probability that the transcribed dNTP to be positioned at *m* when RNAP is fed back. The stochastic entropy production in the entire system can be written as:

(15) $${s^{tot}}_{m}={\;i}_{m}-{\;i^{\prime }}_{m}+∆s(m)$$

and summing for total step *m.* Therefore, when considering the differential in entropy, it follows from the entropy production that Δ*S^tot^_m_* ≥ 0, we have [5,26]:

(16) $$∆s(m)\;\geq -\left(I_{m}-{\;I^{\prime }}_{m}\right)$$

### 2.3. Free Energy Change during RNAP Translocation

Finally, we consider the upper limit of the movement work performed on RNAP. The change in ensemble free energy, represented as the average of Δ*G*(*m +* 1) − Δ*G*(*m*), is denoted as 〈ΔΔ*G_m_*〉. Let *w_m_* represent the work done to DNA at *x*(*t*) by RNAP during the feedback step of the mutual information. Following Equation (16), 〈*w_m_* − ΔΔ*G_m_*〉*β* ≥ 〈*s_m_*〉 − *I*′*_m_*_′_ ≥ − (*I_m_* − *I*′*_m_*) − *I*′*_m_* = −*I_m_* is established. If the work done to RNAP by DNA is *w_m_^ext^* = −*w_m_*, where *w_m_^ext^* represents the external work to RNAP by DNA, then:

(17) $${w_{m}}^{ext}\leq -\langle \Delta \Delta G_{m}\rangle +k_{B}TI_{m}$$

An essential point to note is that, during each cycle of reading a single base pair, the only change in the transcription complex is the movement of RNAP along the template DNA by one base pair, with no other changes occurring within the transcription system. Consequently, it can be stated that the change in free energy due to factors other than RNAP’s movement is essentially zero. Thus, the energy required for RNAP to move must be supplied by some means. Our contention is that the mutual information derived from the base sequence of the template DNA should serve as the source of energy for RNAP’s movement. This theory relies profoundly on the fact that DNA is structured as a repeating double helix and models the movement of RNAP almost as a Markov process. This implies that the type of base read by RNAP does not affect the subsequent reading process.

This conceptualization of free energy accounts for the variations in free energy values reported in experimental studies [18,19,21]. The mutual information per nucleotide read by RNAP from the DNA template is calculated as *I* ≈ −*k_B_T* log (1/4) ≈ 1.4 *k_B_T*. Subsequently, we introduce the conversion efficiency parameter, *α*, which quantifies the proportion of the information gained converted into the mechanical work of forward translocation. The value of *α* ranges between 0 and 1, where *α* = 1 signifies the theoretical maximum efficiency, denoting the complete conversion of information into mechanical work. The work required for the movement of RNAP, considering the effects of mutual information, can be modeled as follows:

(18) $${w_{m}}^{ext}=-\langle \Delta \Delta G_{m}\rangle +\alpha_{m}k_{B}TI_{m}$$

Taking into account the efficiency of mutual information, *I*, on the movement of RNAP, and based on experimental data, the left side is found to be 1.38 *k_B_T* [17,18,19,20], thus estimating *α* to be approximately 0.84. This indicates a high efficiency in converting mutual information into work, implying that the utilization of mutual information in the movement of RNA polymerase is highly effective, underscoring the importance of the role of information in the transcription process. The coefficient *α_m_* is influenced by various environmental factors, making theoretical estimation challenging. Therefore, it should be determined experimentally.

Here, if we denote the entire right side of Equation (18) as −〈ΔΔ*G_m_*′〉, then 〈ΔΔ*G_m_*′〉 − 〈ΔΔ*G_m_*〉 becomes equal to −*k_B_TI*. In this way, by considering the free energy based on ΔΔ*G_m_*′ and taking into account the mutual information *I* obtained by RNAP from the template DNA, it can be said that the free energy increases by *k_B_TI*.

### 2.4. A Proposal for the Information Thermodynamics of Nanopore Sequencing

Employing the principles of information thermodynamics to technologies like nanopore sequencing could improve the accuracy of sequence identification and enrich our comprehension of the process [27]. The data analysis involved in nanopore sequencing necessitates the discernment of valuable information amidst substantial noise. Consequently, a better grasp of the interplay between the quantity of information and the associated noise, guided by the principles of information thermodynamics, has the potential to refine data analysis algorithms.

The configuration of the motor protein and the nanopore located on the membrane within the sequencing apparatus is depicted in Figure 4 [23,24,27,28]. An ionic current traverses the nanopore due to a consistent voltage applied across the membrane, generating a positive charge on the trans side. A motor protein that has helicase activity facilitates the initial unwinding of a double-stranded DNA molecule (or an RNA–DNA hybrid duplex), a critical precursor step, since the nanopore is only capable of accommodating single-stranded DNA. After this unwinding, a single-stranded DNA or RNA, which bears a negative charge, is progressively propelled through the nanopore by the applied voltage. As nucleotides pass through the nanopore, they induce a specific alteration in the current, which enables the determination of the individual nucleotide type at an approximate rate of 450 bases per second [23,29,30,31]. This process corresponds to the drift velocity, *μ*.

It is crucial to emphasize that the movement mechanism to the nanopore reflects the relative motion between the DNA motor protein and the DNA itself. This is a fundamental aspect observed in both the RNAP transcription system and the nanopore sequencing system, wherein the regulated passage of nucleic acids and the sequential identification of nucleotide bases are vital operations. These systems exemplify the sophisticated strategies employed to manipulate biological molecules [30]. Significantly, after the recognition of a single nucleotide, these proteins are reset to their pre-recognition state.

A comprehensive account is necessary to develop the information thermodynamics for nanopore sequencing systems, which encompasses electrochemical effects [21]. This account must detail the variations in the directional movement of nanopore motor molecules along DNA. In this nanopore motor molecule, the relative motion of the molecular motor is directed forward due to the stable potential across the membrane, allowing for the omission of fluctuations in the forward and backward directions. Consequently, the term including *μ/D* in Equation (6) can be omitted, as it can be approximated as follows:

(19) $$∆S\approx k_{B}\log\frac{R_{f}\left(m\right)}{R_{b}\left(m\right)}$$

(20) $$∆s={k_{B}L}^{-1}\log\frac{R_{f}\left(m\right)}{R_{b}\left(m\right)}$$

For the sake of simplicity in the following discussion, we will ignore the dependency of the position *m* on the template DNA in nanopore molecular motors. Furthermore, from Equation (11), $\langle ∆∆G\rangle$ is given as:

(21) $$\langle ∆∆G\rangle \approx -k_{B}T\langle L^{-1}\log\frac{R_{f}\left(m\right)R_{b}\left(m+1\right)}{{R_{f}\left(m+1\right)R}_{b}\left(m\right)}\rangle$$

The external work performed on the motor molecule, indicated by the electrochemical work term, *eV*, using the logarithm of *ϕ* that represents the electrochemical factors to the Gibbs’ free energy [32], is given as follows:

(22) $$eV=-\langle ∆∆G\rangle +{\alpha_{1}k}_{B}TI+k_{B}T\log\phi$$

*α*_1_ denotes a conversion parameter. For simplicity, the mutual information and the electrochemical factor *ϕ* are assumed to be independent of *m*. Experimental observations reveal that when a load voltage of 180 mV is applied during nanopore sequencing, it corresponds to a reading error rate of approximately 38%, as supported by references [27,30,32,33,34]. Consequently, Equation (22) determines the coefficient *α*_1_ as 1 − 0.38 = 0.62. At this membrane potential load, a single nucleotide is observed to simultaneously translocate through the nanopore. Under a higher load voltage of 300 mV [32], potentially leading to reducing the fluctuation in movement independent of *m*, i.e., $R_{f}\left(m\right)\approx R_{f}\left(m+1\right)$ and $R_{b}\left(m\right)\approx R_{b}\left(m+1\right)$. This results in 〈ΔΔ*G*〉 ≈ 0 in Equation (21). (See Equation (10)). When the potential *V* is set to 180 mV or 300 mV, the values of *eV*/*k_B_T* are calculated to be 7.0 (=*v_1_*) or 11.7 (=*v*_2_), respectively [21]. Equation (22) can be rewritten as:

(23) $$v_{1}k_{B}T={\alpha_{1}k}_{B}TI+k_{B}T\log\phi$$

At a higher applied load voltage of 300 mV, it is observed that two nucleobases can pass through the pore. This underscores the significant impact of voltage conditions on the accuracy of nucleobase translocation, when the conversion parameter is set to *α*_2_, as demonstrated in the equation below:

(24) $$v_{2}k_{B}T={\alpha_{2}k}_{B}TI_{m}+k_{B}T\log\phi \prime$$

From Equations (23) and (24), the following result is yielded:

(25) $$\left(v_{2}-v_{1}\right)k_{B}T=k_{B}T\left(\alpha_{2}-\alpha_{1}\right)I-k_{B}T\log\phi \prime /\phi$$

For example, we can obtain the ratio *ϕ*′/*ϕ* by substituting *v*_1_ = 7.0, *v*_2_ = 11.0, *I* ≈ −log (1/4) ≈ 1.4, *α*_1_ = 0.62, and *α*_2_ = 1.0 into Equation (25):

(26) $$\phi \prime /\phi \sim e^{-4.2}$$

As a result, the environmental factor decreased by a factor of *e*^−4.2^ = 0.014. This suggests that the application of a 300 mV voltage is effective in sufficiently reducing environmental factor.

Next, it has been reported that the introduction of polyethylene glycol (PEG) into the reaction systemcan significantly reduce reading errors by crowding effects [33]. To delineate this phenomenon, we first describe the relationship between work, free energy, and mutual information from Equation (22), as follows:

(27) $$v_{1}k_{B}T={\alpha_{3}k}_{B}TI+k_{B}T\log\phi^{\prime \prime }$$

Herein, the addition of PEG to the measurement system results in the environmental factor being amended from *ϕ* to *ϕ*″. From Equations (23) and (27), we derive:

(28) $$0\sim (\alpha_{3}-\alpha_{1}{)\;k}_{B}TI+k_{B}T\;log\frac{\phi^{\prime \prime }}{\phi }$$

And we obtain Equation (29) by substituting *α*_1_ = 0.62, *α*_3_ = 1.0, and *I* = 1.4 into Equation (28):

(29) $$-0.53k_{B}T\approx k_{B}T\;log\frac{\phi^{\prime \prime }}{\phi }$$

This ratio indicates that the addition of PEG reduces the influence of environmental factors or noise by a factor of *ϕ*″/*ϕ*, which is equivalent to e^−0.53^ ≈ 0.59. Thus, the method based on information thermodynamics presented herein enables a quantitative estimation of crowding effect that affects the motion of motor proteins on template DNA. In conclusion, the fundamental equation of Nanopore sequencing is given by Equation (30):

(30) $$eV={\alpha k}_{B}TI+k_{B}T\log\phi$$

## 3. Discussion

This article presented information on the thermodynamics of transcription and nanopore sequencing systems. The informational thermodynamics of RNA polymerase (RNAP) enabled the conceptualization of the transcriptional complex as a so-called Szilard Engine model, capable of converting information into work [5,33,34]. Various experiments have supported the conversion of mutual information into physical work [35,36]. For example, in a specific experimental setup, periodic electron potentials were observed to interact with Brownian particles. The introduction of an appropriate feedback controller into the system prevented the particles from falling back, thereby enabling their transition to higher potential levels [36]. As a result, mutual information could be converted into physical work [36,37,38,39,40].

In contrast to the spontaneous release of energy through the hydrolysis of high-energy phosphate bonds during the formation or elongation reactions between ribonucleotides and deoxyribonucleotides, experimental observations have demonstrated that the movements of RNA polymerase (RNAP) and nanopore motor proteins are governed by stochastic processes. Therefore, the concept of utilizing nucleic acid sequence information to diminish fluctuations in forward motion holds significant implications. The acquisition of information from nucleic acid sequences can reduce the randomness associated with the movements of RNAP, thereby decreasing unnecessary backtracking and the incorporation of incorrect bases during transcription, which, in turn, enhances transcription efficiency. This reduction in energy wastage has the potential to improve the overall efficiency of biological systems.

In the domain of information thermodynamics, the mechanical work derived from “information acquisition” is proportional to the amount of information. The work derived using the mutual information can be expressed as *k_B_TI* and the efficiency of this utilization has been denoted by *α*. The further advancement and refinement of this theory would greatly benefit from detailed and quantitative experimental validation. Specifically, exploring the interactions between enzyme kinetics and nucleotide recognition within nanopores is expected to reveal new insights into the fidelity and efficiency of sequencing.

However, it is important to note that the discussion presented here is not universally applicable to all molecular motors. This discourse is not about perpetual machines that can indefinitely extract mutual information from a thermal bath and convert it into work. Instead, it emphasizes that, in the cases of RNAP and nanopore molecular motors, the focus is on their interaction with evolutionarily refined DNA as the carrier of information and their exposure to the informational chemical bath constituted by deoxyribonucleotides. Furthermore, as mentioned earlier and at the risk of being repetitive, it is essential to note that DNA possesses repetitive structures and with each reading of a single nucleotide sequence by RNAP or nanopore molecules, the thermodynamic state is reset, rendering the system Markovian. This aspect is a crucial condition for the modeling presented herein.

Conclusion: Intracellular molecular motors moving along nucleic acid DNA possess the ability to acquire mutual information from the base sequence and this may be involved in the motor’s own movement. The mechanism by which information is efficiently converted into mechanical work could be a characteristic of biological systems. The model introduced here contributes to the development of methods to mitigate sequencing leader errors, considering factors such as drift speed, diffusion coefficient, and environmental factors in nanopore sequencing, as well as transcription by RNA polymerase.

## Figures and Tables

**Table 1:** Frequency of each mononucleotide in the T7 promoter of the λ phage. 1 ≤ *m* ≤ *N.* If thymine (T) is replaced with uracil (U), the frequency of the occurrence of nucleotides would be the same for both DNA and RNA.

	G	A	T(U)	C	Total
Probability; *P*(*m*)	0.22	0.38	0.16	0.25	1.00
−*P*(*m*) log *P*(*m*)	0.33	0.37	0.29	0.35	1.34
−log *P*(*m*)	1.51	0.96	1.83	1.38	1.42

## Data Availability

All datasets used in the present study are listed in the references.

## Abbreviations

RNAP	RNA polymerase
*m*	Position of the dNTP in the DNA (1 ≤ *m* ≤ *N*)
*L*	The length of the template DNA, equal to *Nd*
*P*(*x*,*t*)	Probability that RNAP is located at any time, *t*, and position, *x*
*P_f_*(*x*,*t*)	Probability distribution of RNAP moving forward at any time, *t*, and position, *x*
*P_b_*(*x*,*t*)	Probability distribution of RNAP moving backward at any time, *t*, and position, *x*
*D*	The diffusion coefficient of RNAP movement
*μ*	Drift velocity of RNAP movement
*R_f_*	Transitional probability in RNAP movement in the forward orientation
*R_b_*	Transitional probability in RNAP movement in the backward orientation
*I_m_*	Mutual information gained by RNAP from dNTP at *m*
*i_m_*	Stochastic mutual information gained by RNAP from dNTP at *m*
*S^tot^_m_*	Total entropy production in the entire system
*s^tot^_m_*	Total stochastic entropy production in the entire system
Δ*s*(*m*)	The entropy produced in the RNAP movement from *m* to *m* + 1 position per length of the template DNA, *L*
*I′_m_*	Mutual information feedback by dNTP at *m*
*i′_m_*	Stochastic mutual information feedback by dNTP at *m*
*w^ext^*	Work done to RNAP at *m* by DNA
Δ*G_m_*	Gibbs’ free energy change before and after RNAP movement
Δ*S*	The entropy produced in the nanopore motor protein movement from *m* to *m* + 1 position
Δ*s*	The entropy produced in the nanopore motor protein movement from *m* to *m* + 1 position per length of the template DNA, *L*
*e*	Electron charge
*V*	Voltage across the membrane in nanopore sequencing
*ϕ*	Electrochemical factors of the entropy
*k_B_*	Boltzmann constant
dNTP	Deoxyribonucleotide
dATP	Deoxyadenosine triphosphate
dCTP	Deoxycytidine triphosphate
dGTP	Deoxyguanosine triphosphate
dTTP	Deoxythymidine triphosphate
rNTP	Ribonucleoside Triphosphates
rATP	Ribonucleoside Triphosphate Adenine
rCTP	Ribonucleoside Triphosphate Cytidine
rGTP	Ribonucleoside Triphosphate Guanine
rUTP	Ribonucleoside Triphosphate Uracil
